# Physiological Mechanisms and Significance of Intracranial B Waves

**DOI:** 10.3389/fneur.2022.872701

**Published:** 2022-05-16

**Authors:** David W. Newell, Maiken Nedergaard, Rune Aaslid

**Affiliations:** ^1^Department of Neurosurgery, Seattle Neuroscience Institute, Seattle, WA, United States; ^2^Department of Basic and Translational Neuroscience, Faculty of Health and Medical Sciences, University of Copenhagen, Copenhagen, Denmark; ^3^Department of Translational Neuromedicine, University of Rochester Medical School, Rochester, NY, United States; ^4^Department of Neurosurgery, University of Bern, Bern, Switzerland

**Keywords:** B waves, transcranial Doppler, intracranial pressure, glymphatic pathway, cerebrospinal fluid circulation, cerebral blood flow, non-REM sleep, delta wave EEG

## Abstract

**Objective:**

Recently published studies have described slow spontaneous cerebral blood flow (CBF) and cerebrospinal fluid (CSF) oscillations measured by magnetic resonance imaging (MRI) as potential drivers of brain glymphatic flow, with a similar frequency as intracranial B-waves. Aiming to establish the relationship between these waveforms, we performed additional analysis of frequency and waveform parameters, of our previously published transcranial Doppler (TCD) and intracranial pressure (ICP) recordings of intracranial B waves, to compare to published MRI frequency measurements of CBF and CSF slow oscillations.

**Patients and Methods:**

We analyzed digital recordings of B waves in 29 patients with head injury, including middle cerebral artery (MCA) flow velocity (FV), ICP, end tidal CO_2_, and arterial blood pressure (ABP). A subset of these recordings demonstrated high B wave activity and was further analyzed for parameters including frequency, interaction, and waveform distribution curve features. These measures were compared to published similar measurements of spontaneous CBF and CSF fluctuations evaluated using MRI.

**Results:**

In patients with at least 10% amplitude B wave activity, the MCA blood flow velocity oscillations comprising the B waves, had a maximum amplitude at 0.0245 Hz, and time derivative a maximum amplitude at 0.035 Hz. The frequency range of the B waves was between 0.6–2.3 cycles per min (0.011-0.038 Hz), which is in the same range as MRI measured CBF slow oscillations, reported in human volunteers. Waveform asymmetry in MCA velocity and ICP cycles during B waves, was also similar to published MRI measured CBF slow oscillations. Cross-correlation analysis showed equivalent time derivatives of FV vs. ICP in B waves, compared to MRI measured CBF slow oscillations vs. CSF flow fluctuations.

**Conclusions:**

The TCD and ICP recordings of intracranial B waves show a similar frequency range as CBF and CSF flow oscillations measured using MRI, and share other unique morphological wave features. These findings strongly suggest a common physiological mechanism underlying the two classes of phenomena. The slow blood flow and volume oscillations causing intracranial B waves appear to be part of a cascade that may provide a significant driving force for compartmentalized CSF movement and facilitate glymphatic flow.

## Introduction

Slow oscillations of cerebral blood flow (CBF) and volume have recently emerged as a topic of interest because these slow oscillations are associated with cerebral spinal fluid (CSF) movement in the brain, and may facilitate flow through the brain interstitial space for clearance of solutes and toxic metabolites in a process termed glymphatic flow (1). Coupled, slow synchronous oscillations of intracranial EEG, MRI blood oxygen level dependent (BOLD) signal, and CSF waves (2), together appear to play a crucial role in driving CSF movement, especially during slow wave (delta wave) sleep activity. Furthermore, these types of oscillations occur in the same frequency range as intracranial B waves, which are also the result of regular synchronous fluctuations in CBF, and intracranial pressure(ICP), and are of unknown origin (3). This association prompted us to analyze additional frequency parameters, and waveform features from previously performed intracranial recordings of MCA velocity and ICP during B waves (3), and compare them to published MRI measurements of CBF slow waves (2, 4–9) to determine the similarities between these entities.

The source of intracranial pressure B waves, originally described as regular repeating ICP oscillations occurring at 0.5–2 cycles per min, has proven elusive, and their physiological role has not been established. Lundberg commented in his original and classic paper, that examination of the features of B waves, and their relationship to other physiologic parameters, permitted no definite conclusion as to their origin (10). An observational study of pial arteries in anesthetized cats described synchronous ICP waves and vascular diameter fluctuations, which occurred at a similar frequency (0.5–2 per min) as classical B waves, supporting the notion that cyclic blood flow and blood volume fluctuations were the likely cause of ICP B waves, but did not give any indication of their physiological function (11).

Several early reports of transcranial Doppler (TCD) ultrasound recording in patients and normal subjects, described middle cerebral artery (MCA) velocity fluctuations occurring due to CBF changes at the same frequency range as Lundberg B waves (12, 13). We reported MCA velocity fluctuations in the same frequency range (0.5–2 per min) and of a similar form, as Lundberg B waves in 70 % of normal subjects who were resting and lying on a stretcher for 1 hour and in the same report, described synchronous MCA velocity and ICP oscillations at the same frequency as B waves in head injury patients (3).

Other investigators have confirmed these results, and further characterized rhythmic oscillations in the MCA flow velocities in a variety of settings, including head injury patients, in normal resting volunteers, and also during sleep (14–18). Several studies noted that B waves measured by TCD occur over a wider frequency range than Lundberg noted in ICP recordings, and over a wider frequency that our group had originally described (3), leading to the proposal that the B wave frequency range be extended to 0.33–3 cycles per min (0.005–0.05 Hz) (18). Other investigators have reported intracranial B waves occurring at frequencies as high as four cycles per min (0.067 Hz) (19).

More recently published descriptions of slow periodic CBF oscillations measured by functional (f)MRI, coupled to EEG and CSF fluctuations in the same frequency range as B waves, strongly indicate that these coupled oscillations are metrics of the same physiological process as B waves, without having been recognized as such (2, 4, 5, 7–9). To explore this possibility, we analyzed additional parameters of stored digital recordings of B waves from head injured patients (3), aiming to compare their frequency, wave properties, and interaction, to those of the more contemporary MRI results showing slow CBF and CSF oscillations at a similar frequency. We also compared these newly analyzed results, to those described in other review articles of intracranial B waves, which included published frequency values.

## Materials and Methods

### Data Analysis Methods

The analysis is based on recordings from head trauma patients at the Inselspital ICU in Bern, Switzerland. Continuous recording of arterial blood pressure(ABP), end tidal CO_2_ (Et CO_2_), ICP, was collected, and MCA velocity was also recorded simultaneously for intervals, as part of a study on TCD in head injury. The spectral outline of the MCA velocity signal was taken along with ABP, ICP, Et CO_2_, as analog signals, sampled at 50 Hz, processed through an analog to digital converter, and stored digitally on a personal computer. Patients were managed according to accepted standards for moderate and severe head injury, including early intubation, early surgical evacuation of mass lesions, monitoring and control of ICP using sedation, paralytics, moderate hyperventilation, and osmotic diuretics. Monitoring of ICP was accomplished using surgically implanted epidural pressure monitors (manufactured by Gaeltec, Ltd., Dunvegan, Scotland). In this original publication (3), we defined B waves, by manual counting, as at least 2 min of ICP and MCA velocity waves, at a frequency of 0.5–2 per minute as defined by Lundberg (10).

The digital data, including 262 recordings from 29 patients (Table 1), were converted into an up-to-date floating decimal point digital format. Some recordings had insufficiently long (<500 s) contiguous intervals without artifacts or interventions such as respirator changes or ABP manipulation; these recordings were not included for further analysis. Because the epidural ICP probe gave unreliable pulsatile readings for very low absolute values, we also eliminated recordings with a mean intracranial pressure (mICP) of <5 mmHg. TCD recordings of abnormally low mean flow velocity (mFV <20 cm/s) were also rejected from consideration in the final analysis. In the remaining 151 recordings (from 26 patients), we selected the longest available segment without artifacts or interventions for further analyses. A separate heart rate (HR) data record was calculated from the timing of upstrokes in the ABP waveform data.

**Table 1:** Overall patient characteristics (*n* = 29) and physiological data.

Age;	Average = 34	Range = 16–70	
Gender	Males = 22	Females = 7	
Admission Glasgow Coma Score*	Average = 5.7	Range = 3–9	
Physiological variables (Mean ±S.D.)			
B-wave amplitude (MCA velocity)	Low <5%	Median 5–10%	High > 10%
ABP (mmHg)	84.1 ± 8.8	88.5 ± 9.9	91.2 ± 10.6
Heart Rate (beats/min)	88.1 ± 12.7	87.1 ± 12.6	88.7 ± 12.3
EtpC0_2_ (mmHg)	29.2 ± 5.5	29.9 ± 6.7	32.3 ± 7.2
ICP (mmHg)	14.9 ± 7.6	13.9 ± 5.3	15.3 ± 5.8
MCA flow velocity (cm/s)	49.1 ± 15.1	47.2 ± 17.6	46.3 ± 13.1
Number of recordings	18	23	13
Number of patients	68	52	31

* *Available on 27 patients*.

Heartbeats, respiration and induced random oscillations were attenuated by a 0.12 Hz sixth order Butterworth low-pass filter. Multiple discrete Fourier transforms of the samples were made with an overlap of 87.5% and a resolution of 0.0025 Hz. A Blackman-Nuttall window was used to reduce spectral leakage. The means of the amplitude and power spectral densities for all overlapping transforms were calculated. The B-wave power was calculated as the sum of the power spectral densities of frequency bins in the range of 0.01 to 0.06 Hz, and the B wave amplitude (BWA) was defined as the square root of the B wave power. We note that this definition differs from the usual peak-to-peak definition; for a purely sinusoidal wave, the peak-to-peak height would be twice that of the amplitude determined by spectral analysis. The B wave amplitudes were calculated in conventional units (mmHg and cm/s) and also as a percentage of the mean values of the variables over the selected intervals.

### Formulas and Statistics

**Figure 1:**
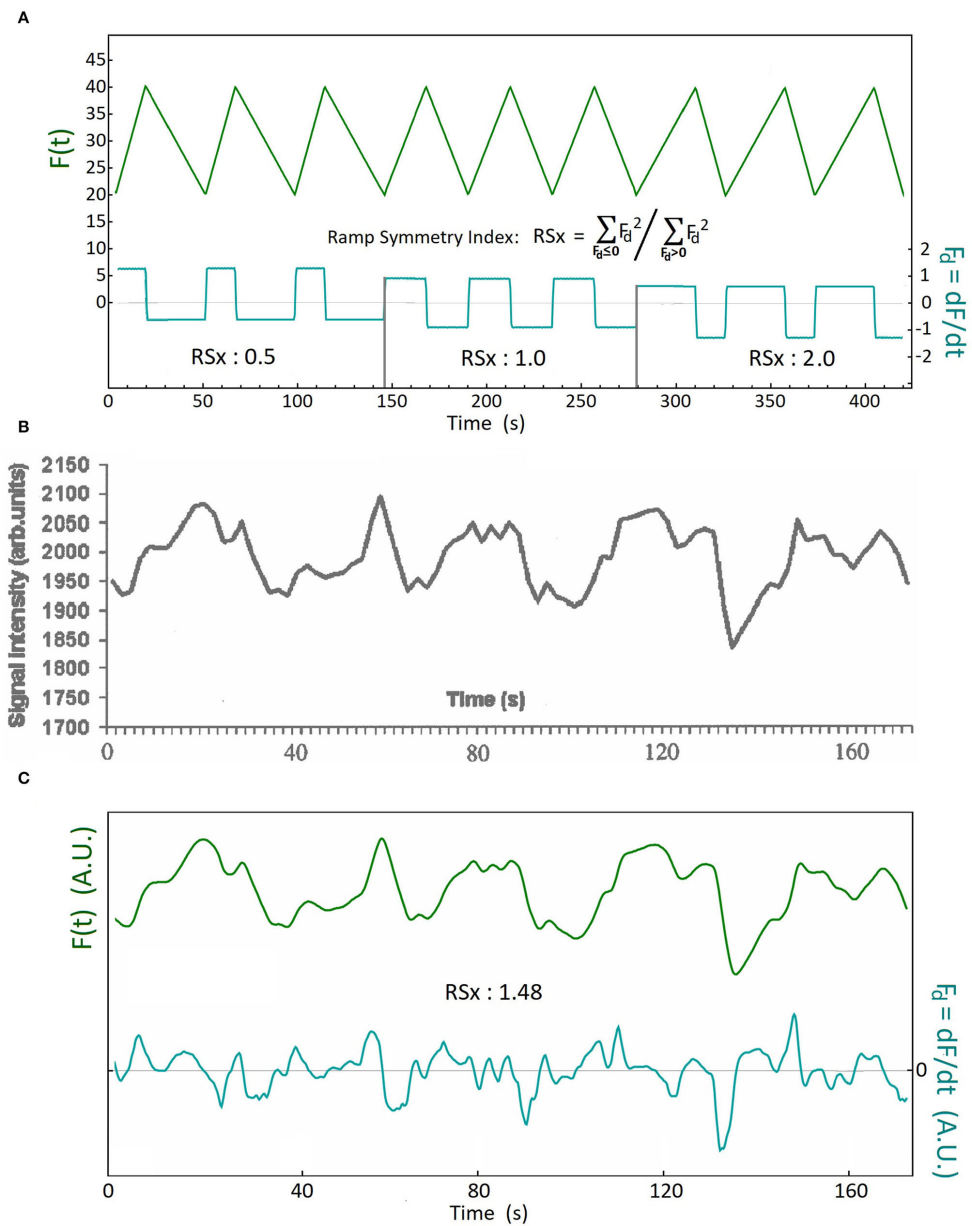
The definition of the Ramp Symmetry Index (RSx), and an illustrative example using analysis of a CBF slow wave. **(A)** Upper tracing: F(t) is a ramp function with three different rates of descent and ascent. This is manifest in the time derivative F_d_(t) of the function (lower tracing). The time derivative time series is used to calculate the ramp symmetry index RSx. It is defined as the ratio of the sum of all squared samples with a negative sign (before squaring) divided by the sum of all squared samples having a positive sign. (see methods for formula); **(B)** An example of a continuously displayed blood oxygen level dependent (BOLD) MRI signal sequence, reflecting CBF slow oscillations, as reported by Kiviniemi et al. (20) (reproduced with permission); **(C)** The upper tracing (*green*) is the digitized and low-pass filtered tracing of the published BOLD signal in **(B)**. The lower tracing (*cyan*) is the time derivative of the upper. The ramp symmetry index of the sequence is calculated using the formula in **(A)**. An RSx of 1.48 means that the rate of decent is on average higher than the rate of ascent in this example. A plateau shaped wave is demonstrated by the third wave at the 80 second mark, and a double peaked wave is demonstrated by the fifth wave, at the 160 second mark [tracing **(B)**, and upper tracing in **(C)**].

We performed a ramp symmetry index (RSx) as described in Figure 1 to quantify this property, and for purposes of comparison to published fMRI continuous CBF tracings. This index quantifies how much faster the rate of descent is, compared with the rate of ascent.


$$\begin{array}{l} \text{Formulas}:\;{\text{F}}_{\text{d }}=\text{dF}(\text{t})\text{/dt},\\ \text{ RSx}=\frac{\sum_{{\text{F}}_{\text{d}}<0}{\text{F}}_{\text{d}}^{2}}{\sum_{{\text{F}}_{\text{d}}>0}{\text{F}}_{\text{d}}^{2}} \end{array}$$

Statistical analysis. For group comparisons we used a Student's *t*-test and considered significance of *p* < 0.05.

## Results

### Wave Analysis Results

The individual waves had different heights and lengths (periods), and sometimes also occurred as sinusoidal, ramp shaped, wave doublets, or as plateau like shapes, as has been previously described (19). The discrete Fourier transform gives a good quantitative representation of the frequency of the B waves but is not descriptive of the shape and form of the individual waves. The results of simple inspection of these waves, indicates that the transition from the crest to the trough is briefer and steeper than the transition from trough to crest.

**Figure 2:**
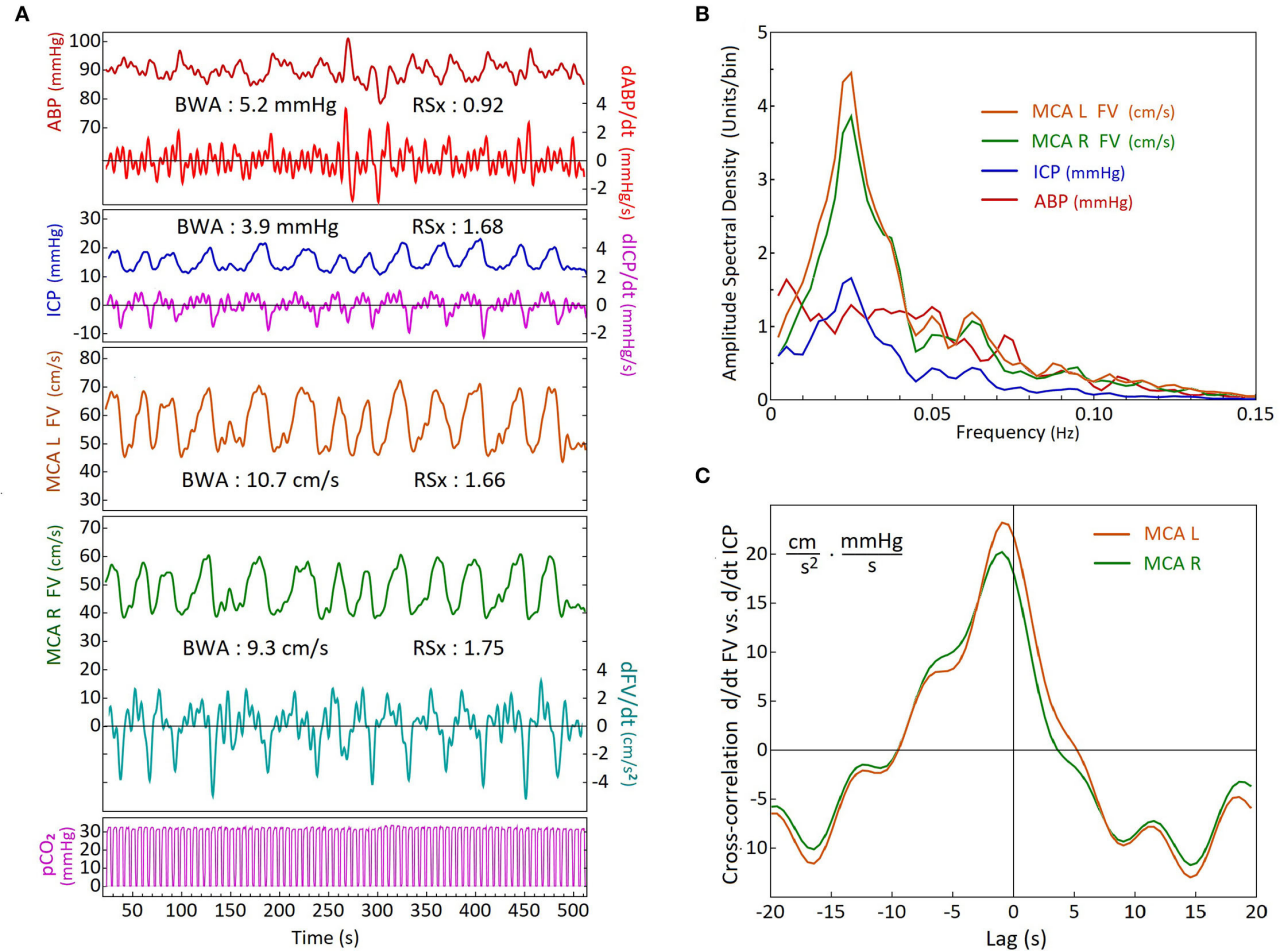
Comparison of activity and timing of simultaneous blood pressure, flow velocity, intracranial pressure and end tidal CO_2_ recordings during B waves, using frequency analysis and cross-correlation. **(A)** Time-series recordings from a head trauma patient on Day 5, showing arterial blood pressure (ABP), intracranial pressure (ICP), bilateral middle cerebral artery (MCA) flow velocities (FVs), and airway partial CO_2_ pressure (pCO_2_). The time-derivative series of Right FV, ICP and ABP are also shown. The ramp symmetry indices (RSx's) were calculated from the time-derivative series (labels on right). Note that the ICP and the FV's have similar waveform asymmetry and higher Rsx values > 1(faster down than up) whilst the ABP RSx indicates slightly faster up than down with an Rsx value <1. A double peak wave morphology is demonstrated by the right MCA flow velocity tracing in green between the 200 and 250 second mark. (R, right; L, left); **(B)** Results of spectral analysis using discrete Fourier transforms of the time-series data in **(A)**. The amplitude spectral density curves are similar for the FVs and the ICP, showing distinct peaks within the B wave frequency range. The ABP amplitude spectral density curve has a different shape with a much flatter distribution over most of the frequency range (as in the composite of the group shown in Figure 3A); **(C)** Cross-correlation analysis of dFV/dt versus dICP/dt. There is only a small difference in amplitude between the sides which is probably due to the mean FV of the left MCA being slightly higher. For both sides, the dFV/dt leads the dICP/dt by approximately 1 sec.

The results of a waveform asymmetry analysis using the RSx, of the linear interval of an fMRI BOLD recording during slow CBF oscillations, published by Kiviniemi et al. (20), is shown as an example in Figure 1 (with permission). If the CBF oscillations were sinusoidal, we would expect an RSx close to one. The ramp symmetry index as calculated in Figure 1C greatly exceeds unity, due to the predominance of waves with descent rates exceeding the rate of ascent, which is similarly observed in intracranial B waves (Figure 2). We also found additional figures published in two other articles (2, 9) which provided continuous tracings of fMRI BOLD signals during CBF slow oscillations and permitted the calculation of RSx values. These values were then compared to the RSx values of the CBF slow wave study referred to above, and also to the RSx values from our B wave analysis. Additional details of the frequency values and other parameters from these publications are given in Table 2.

**Table 2:** Human studies comparing frequency measurements of slow wave CBF and CSF oscillations using MRI imaging (upper) *vs*. analysis of TCD derived CBF, and ICP pressure measured oscillations (lower) cpm, cycles per min; Hz, cycles per sec.

**Author Method**	**CBF frequency range**	**CBF frequency peak mean**	**CSF**	**EEG**
Horowitz et al. (8) MRI Bold (sleep)	0.01–0.03 Hz, 0.6–1.8 cpm	0.02–0.03 Hz 1.2–1.8 cpm	No	Yes
Fukunaga et al. (7) MRI Bold (sleep)	0.01–0.05 Hz 0.6–3 cpm		No	Yes
Raichle and Snyder (9) MRI Bold (resting)	Not given	Approx 2–3 cpm	No	No
Fultz et al. (2) MRI Bold and CSF (sleep)	Not given	d/dt max=0.05 Hz or “about” 3 cpm	0.05 Hz or 3 cpm in 4th ventricle (peak)	Yes
Kiviniemi et al. (4) MRI Bold, (anesthesia)	0.025–0.041 Hz 1.5–2.5 cpm	0.033 Hz 2.0 cpm		No
Kiviniemi et al. (6) MRI Bold (sedation)	0.02–0.06 Hz 1.2–3.6 cpm			No
Kiviniemi et al. (5) MRI CSF pulsations in brain parenchyma	**Total range** LF= low frequency VLF= very low frequency		**1.6**–**4.4 (cpm) 0.01**–**0.073 Hz** 1.6–4.4 (cpm) 0.027–0.073 Hz 0.61 = 1.6 (cpm) 0.01–0.027 Hz	
**Current findings TCD and ICP** (head injury)	0.011–0.038 Hz 0.6–2.3 cpm	Mean= 0.0245 Hz, 1.5 cpm	ICP monitoring	No
Spiegelberg et al. (18) **TCD and ICP** (review)	0.0055–0.05 Hz 0.33–3 cpm		ICP monitoring	No
Martinez-Tejada, et al. (19) **TCD and ICP** (review)	0.003–0.067 Hz 0.2-4 cpm		ICP monitoring	No

The results of the ramp symmetry index calculations of slow CBF oscillations measured using digitized scanned figures of the fMRI from the three articles (2, 9, 20) which contain linear BOLD tracings, revealed RSx values of 1.58, 1.48, and 1.45 respectively. The results indicate a marked asymmetry and ramp like configuration of the slow CBF waveform compared to a pure symmetrical wave which has an RSx value of 1.0. The average RSx value calculated from the CBF oscillations in these three articles equals 1.50 (*n* = 3) vs. an average RSx value of 1.49 (*n* = 31) in the flow velocity values in the high B wave amplitude group.

Comparison of the CBF slow wave tracing shown in Figures 1B,C can be made to our flow velocity (FV), and ICP B wave tracings, in Figure 2A as examples. The FVs and ICP waves have clear wave asymmetry, with RSx values of right MCA FV=1.66, left MCA FV=1.75, and ICP=1.68, respectively. The ABP waves were practically symmetrical in shape, with an RSx of 0.92. The spectral analyses of these tracings are shown in Figure 2B. Both FV tracings, as well as the ICP, have maxima at a frequency of 0.025 Hz. The ABP also exhibits moderate fluctuations, but without showing any distinct maximum in the B wave frequency range. The EtCO_2_ reflects the constant frequency signal of mechanical ventilation. Thus the FV and ICP B wave fluctuations appear not to be caused by similar changes in ABP and CO_2_. The cross-correlation analysis of d/dt FVs vs d/dt ICP is shown in Figure 2C; we see that the dFVs are preceding the dICP by about 1 s, which is nearly equal on both sides (R and L MCA velocity). (The undulations in the cross-correlations are due to a relatively slow respiration rate for this patient).

### Wave Comparative Analysis

High B-wave activity, defined as a flow velocity B wave amplitude exceeding 10%,medium B-wave activity, with B wave amplitude between 5 and 10%, and low B wave activity with B wave amplitudes below 5%. The overall patient data, and physiological findings in these three B wave activity groups are listed in Table 1.

**Figure 3:**
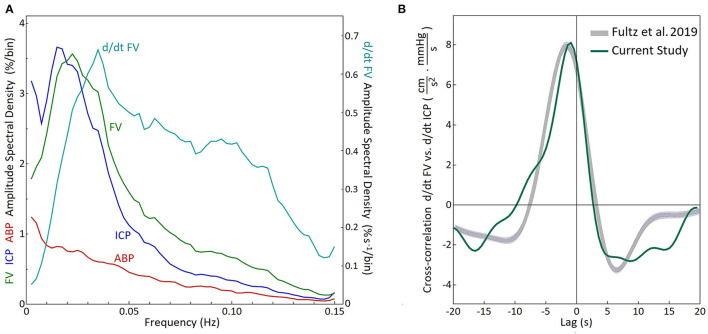
Analysis of recordings of the patient group with high B wave activity. **(A)** Composite of discrete Fourier transform spectral analysis of 31 recordings with high B wave activity (B wave amplitude above 10% of mFV) in 13 patients. The composite amplitude spectral density was calculated as the square roots of the averaged power spectral densities for each frequency bin. Maximum spectral density was found at 0.0225 Hz for the FV and 0.0175 Hz for the ICP. The time derivative for the FV had a maximum amplitude at 0.035 Hz (d/dt FV) FV, flow velocity; ICP, intracranial pressure; ABP, arterial blood pressure. **(B)** Composite cross-correlation of d/dt FV vs. d/dt ICP for the same 31 recordings (Green). The gray curve shows the cross-correlation (-d/dt BOLD vs. CSF movement) reported by Fultz et al. (2) during sleep in normal subjects. (The curves share same zero line and the arbitrary units used for the MRI data have been scaled to facilitate comparison.)

The composite spectral analyses of all recordings with high B wave amplitude are shown in Figure 3A. Normalization with respect to the mean values resulted in similar amplitudes and frequency dependency of the spectral densities for the FV and the ICP. In particular, maximum spectral density the flow velocity signals (cyan line), which had a peak at 0.035 Hz. In Figure 3B we present a direct comparison of the composite cross-correlation results (d/dt FV vs. d/dt ICP) in these recordings, with the cross-correlation results of d/dt MRI BOLD vs. CSF movements in the 4^th^ ventricle as reported by Fultz et al. (2). The curves are quite similar in timing and form, supporting the notion that a similar cause and effect relationship may exist between MRI measured BOLD vs CSF movement, as exists between TCD measured FV vs ICP change, although the fMRI cross-correlation was restricted to unipolar CSF and –d/dt BOLD signals.

**Figure 4:**
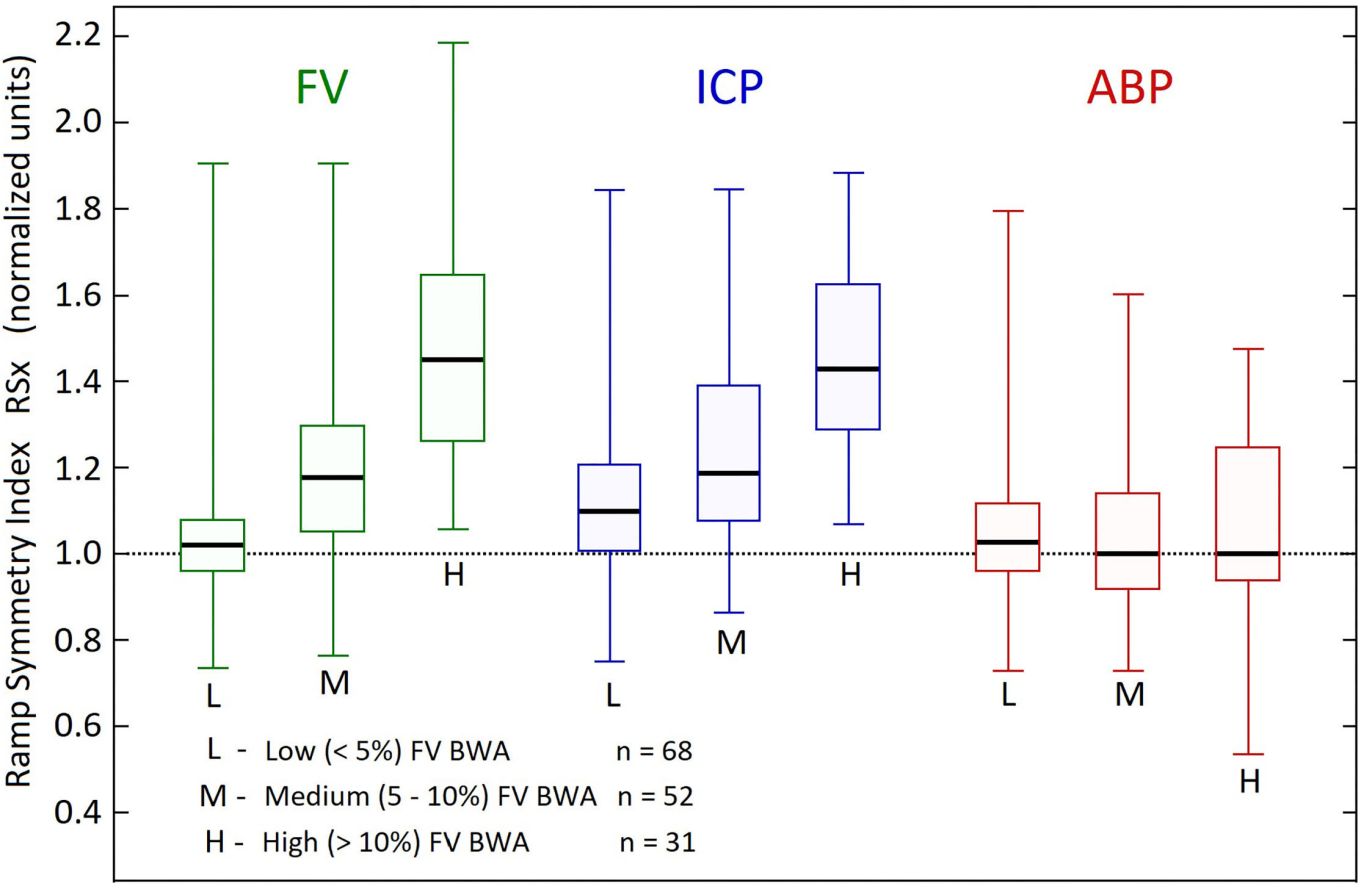
Comparison of ramp symmetry index across B wave activity groups. Box plots of the distribution of ramp symmetry indices (RSx) in the three B wave activity (BWA) groups according to B wave amplitude, related to arterial blood pressure (ABP), intracranial pressure (ICP), middle cerebral artery flow velocities (FV). The Boxes represent upper and lower quartiles, with the thick horizontal lines within the boxes, showing the medians. The vertical lines with the top and bottom caps, represent maximum and minimum RSx values for each variable and Group. The mean RSx in the High BWA group (FV mean = 1.49, median = 1.45) and also the FV and ICP for high and medium groups significantly exceeded unity (*p* < 0.001, 2 tailed t test). The ABP in the both the high and the medium BWA groups were not significantly different from 1. (*p* = 0.3 and p = 0.07, two tailed *t*-test).

The wave-shapes of FV and ICP become more asymmetric with increasing B wave amplitude, as shown in the box plots in Figure 4. The RSx values in the High B wave activity group (FV mean=1.49, median=1.45) and also the FV and ICP for high and medium groups significantly exceeded unity (*p* < 0.001, 2 tailed *t*-test), indicating a more rapid rate of descent, than ascent. In contrast the value of the RSx of the ABP did not change in a similar manner and the ABP in the both the high and the medium BWA groups were not significantly different from 1. (*p* = 0.3 and p = 0.07, two tailed *t*-test), indicating that the FV and ICP B wave fluctuations were not caused by similar fluctuations in the ABP.

The heart rate data were influenced by arrhythmias in 10 recordings for the low FV BWA group, 5 in the medium; and *non*e in the high group. These corrupted recordings were omitted from further analysis by discrete Fourier transform. The HR BWAs were calculated in the same manner as for the FV, ABP and ICP data. The HR BWAs were low, the group Means ± S.D. were 1.66 ± 1.12%, 1.90 ± 1.42%, and 2.01 ± 1.84% in the low, median and high groups, respectively. Using a two-tailed *T-test* (assuming unequal variance) on the HR BWA data showed no significant difference between groups. The lowest *p-value* was *p* > 0.28 between the low and the high groups.

### Wave Frequency Comparisons to Published Studies

**Figure 5:**
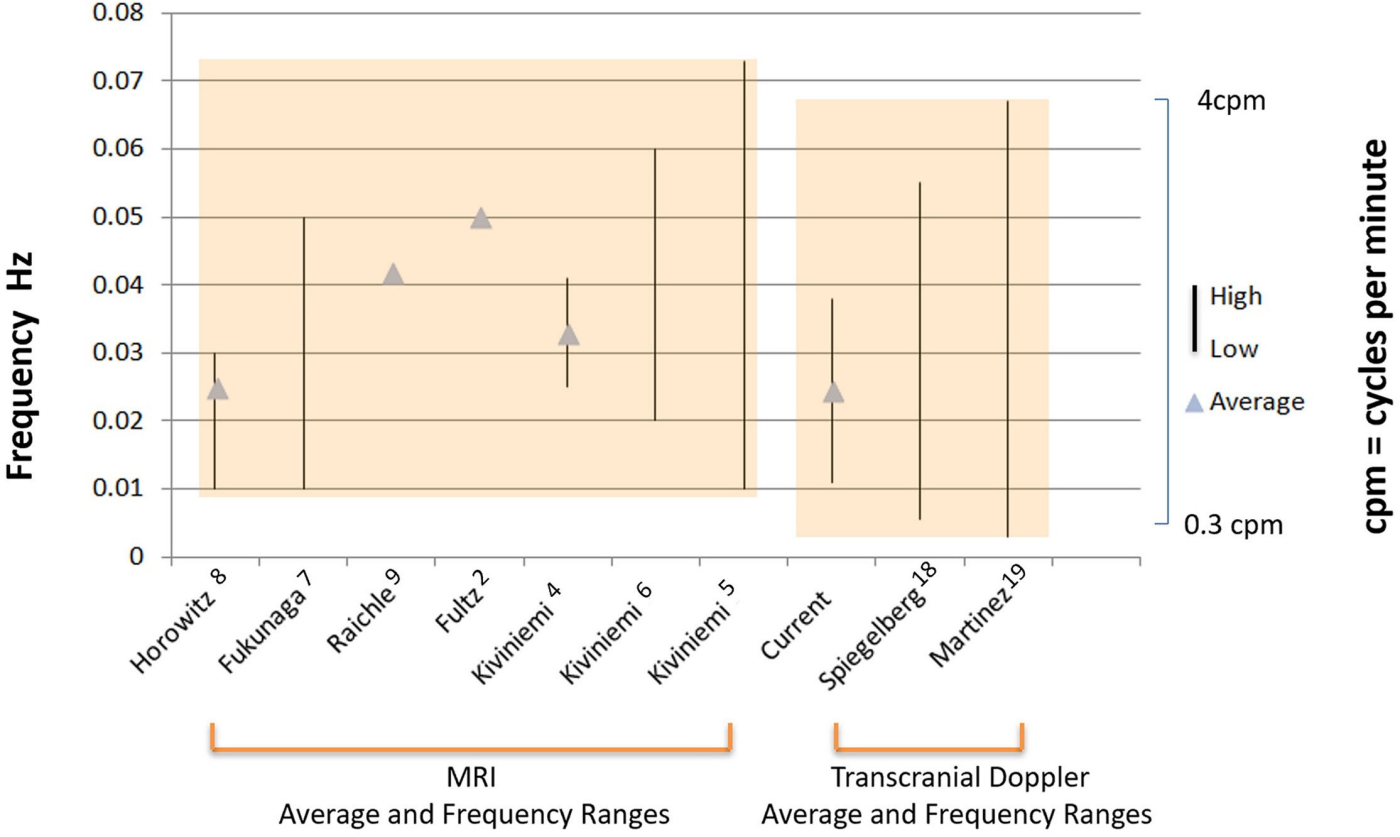
Comparison of MRI and TCD measured frequency values of CBF and CSF oscillations. Illustration of the graphic comparison of the raw numerical data given in Table 2, for visualization of both the individual studies which reported either mean frequencies (triangles), and, or frequency ranges (solid vertical lines) for MRI slow wave oscillations (left bracket), or B waves recorded by TCD and ICP (right bracket). (note: some studies reported mean frequency values only, indicated by triangles, some studies reported frequency ranges only, indicated by vertical lines, and others reported mean and range values for frequency, indicated by both triangles and vertical lines). The first authors of each of the studies and reference numbers are given in the same order from left to right in which they appear in Table 2 from top to bottom. The values for the label current, refer to results of the frequency analysis for B waves presented in this paper. The overall range of values between the MRI derived data, and the TCD and ICP derived data is also indicated by the shaded boxes, which emphasize the close overlap of these group values to each other.

We then compared frequency measures of the B wave values derived from the reanalysis of our recordings to B wave frequency values published in two review articles on B waves, and also to published values of MRI measured CBF slow waves and CSF oscillations, in both the table format, (Table 2) and also as a comparison graph (Figure 5). Table 2 compares our present analysis results to those in relevant published studies of MRI measurements, which included frequency ranges and/or average frequency measurements of CBF and CSF oscillations. Included, are subjects who were asleep or resting, and also patients who were placed under sedation or general anesthesia (left column of table). The frequency values in these published reports are compared to the frequency range and average maximum values obtained from our present analysis, as well as the results from two published review articles of ICP B waves. Figure 5 illustrates the graphic form of these data values, for comparison of the raw data given in the table, and for visualization of group comparisons.

## Discussion

### Historical Aspects, and Comparison of B Waves to FMRI Fluctuations

Lundberg first described distinct types of intracranial pressure waves, termed A, B, and C waves, in an article titled “Continuous Recording and Control of Ventricular Fluid Pressure in Neurosurgical Practice” describing results in brain tumor patients at a neurosurgical intensive care unit in Lund, Sweden (10). The A waves, or plateau waves, were described as sudden pronounced marked increases in ICP, which then formed a plateau pattern for a variable period of time (5–20 minutes), and then decreased sharply at the conclusion of the wave, usually under conditions of severely reduced intracranial compliance.

The B waves were described as regular lower intensity ICP fluctuations, repeating at a frequency of 0.5–2 cycles per min (10), and were subsequently demonstrated to be due to cyclic CBF oscillations (11). Introduction of TCD, allowed continuous measurement of intracranial flow velocity in humans, which reflects blood flow changes from baseline recordings (21, 22). Regular MCA velocity fluctuations, were observed at the B wave frequency range, and were subsequently described in cases of head injury, subarachnoid hemorrhage, hydrocephalus, as well as in normal individuals during deep relaxation and sleep (3, 14, 15, 18).

Lundberg's C-waves reflect brief (approximately 0.1 Hz) irregular fluctuations in arterial pressure brought about by fluctuations in baroreceptor and chemoreceptor reflex control systems, also known as Traube-Hering-Mayer waves in cardiovascular physiology (23).

Regional measurements of cortical metabolic activity for localization of cerebral function using fMRI BOLD sequences have revealed cyclic fluctuations in the baseline values, in the same frequency range as intracranial B waves (Table 2, Figure 5). These spontaneous fluctuations of linear tracings using fast signal processing of BOLD activity, have been referred to as CBF slow waves, and have hitherto been considered an unwanted source of noise and measurement variability, when performing baseline measurements for functional MRI. Their cause has been attributed to factors including fluctuating EEG input, random brain activity, and modulation of brain metabolic demand (9, 24). Moreover, the predominant frequency of these large amplitude spontaneous CBF oscillations was 0.01–0.05 Hz or 0.6–3 cycles per min (7), which is in the same frequency range as cyclic CBF oscillations causing intracranial B waves (18). Obrig et al. (25) have reviewed other continuous brain blood flow and hemodynamic activity measurements, including TCD, fMRI BOLD, laser Doppler, and near infrared spectroscopy, showing oscillations at the same core frequency of intracranial B waves. It is now apparent that these studies are likely describing similar CBF fluctuations over a common frequency range but using different methods.

In addition to sharing similar frequency values, CBF slow waves and intracranial B waves also display common morphological features, including, ramp-like features, and irregularly occurring “plateau like” features, or wave doublets (19). The quantitative analysis of asymmetry performed, in this report, clearly indicated that the ramp features were highly prevalent in the group of B waves analyzed, as evidenced by the mean RSx values of ≥1 (Figures 2, 4). Many of the fMRI-based reports of CBF slow waves have not included raw linear BOLD sequence tracings at the interval required to examine and compare the CBF slow waves for these morphological changes. We found three reports however, with illustrative figures displaying the interval required for analysis from the illustrations, and these reports confirm a ramp-like morphology and irregularly occurring plateau features (2, 9, 20). Quantitative analysis of the RSx values indicating the degree of asymmetry of the CBF slow waves from the illustrated MRI tracings, all three of these reports revealed the calculated RSx values of the CBF slow waves, greatly exceeded 1 (avg RSx = 1.50, see results), similar to the FV values from our B wave analysis (avg RSx = 1.49) (see results, Figure 4). Such findings support the notion that the same physiological generator in the central nervous system, is operating to cause CBF oscillations with similar finer subtle features in wave morphology, including asymmetry, as measured either by TCD, or fMRI BOLD signal linear tracings. It should also be noted that in our study, respiration and arterial pCO_2_ were controlled and practically constant during the analysis. While this does not exclude that respiration may have an effect on B waves in normal spontaneous breathing, the controlled respiratory environment of our patients strongly suggests that B waves are mainly generated by another factor.

### Origin of EEG Oscillations, CBF Slow Oscillations, and Intracranial B Waves

Coupled oscillations of EEG and intracranial B waves, reported in head injury patients (16, 26), during a baseline of delta wave EEG activity, are physiologically similar to the coupled EEG and CBF, and CSF movement patterns observed in healthy subjects during non-REM sleep (2, 8). The method of EEG recording and display used by Lescot et al. (16) in head inured patients, is called EEG spectral outline tracing (27, 28), which displays the highest of multiple EEG frequencies as a single linear envelope. The EEG pattern, which was reported during B wave activity, consisted primarily of delta wave EEG frequency (0.5–4 Hz), with faster theta wave (4–7 Hz) EEG activity episodes, preceding each of the B waves, linked to transient increases in MCA velocity and ICP. This coupled activity between EEG, MCA velocity and ICP, is well illustrated in this publication by Lescot et al. (16). In contrast, EEG spectroscopy (29), is a different method which displays multiple EEG frequencies simultaneously, and continuously. Studies using this method, however, have also shown cyclic faster linked EEG frequency clusters, preceding the MRI detected CBF slow oscillations in human volunteers during sleep, occurring in the same frequency range as intracranial B waves (2, 8).

### Central Pacemaker Role of Deep Brain Structures Including Locus Coeruleus

**Figure 6:**
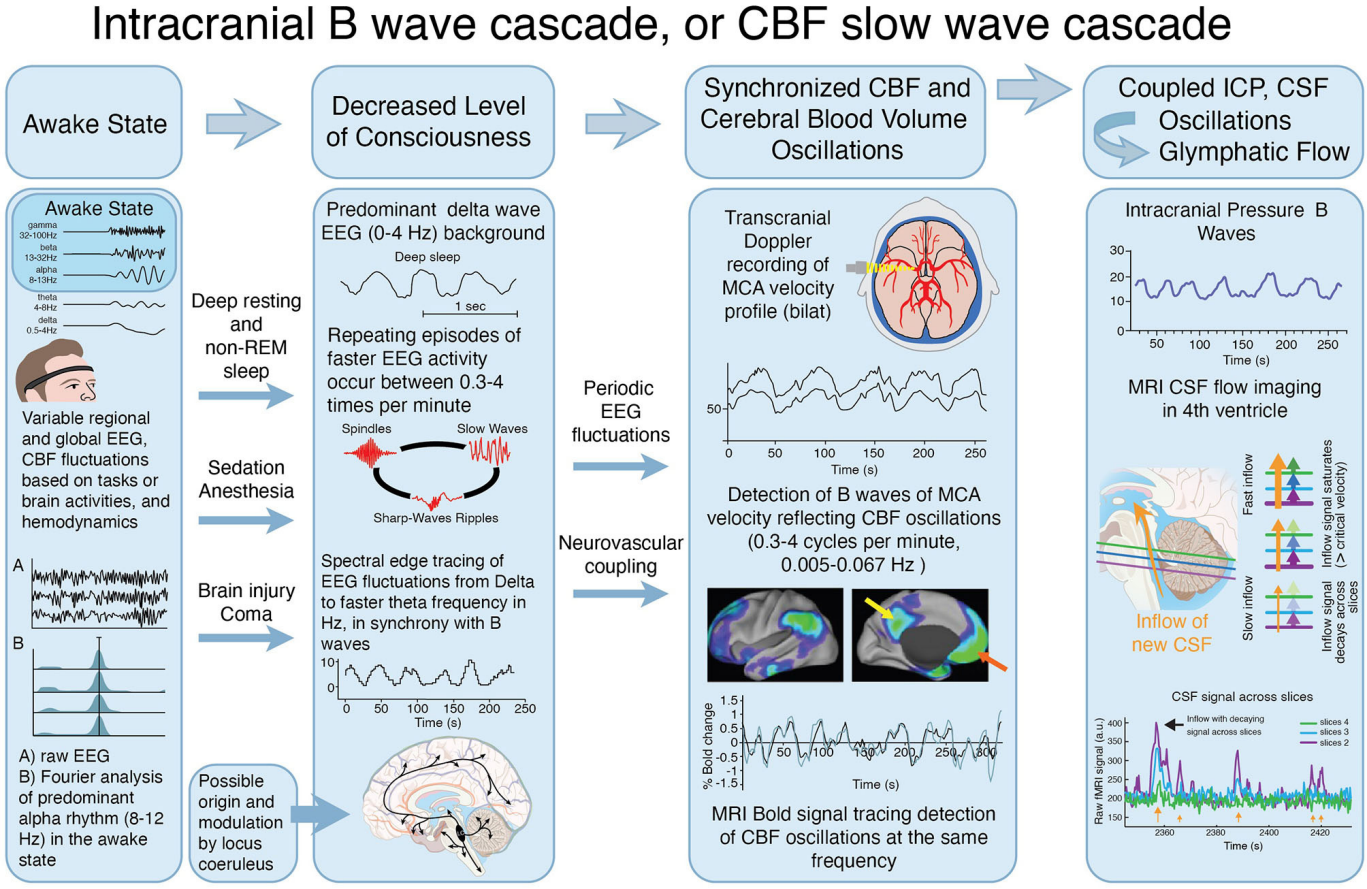
Proposed interactions between clinical state, EEG activity, CBF oscillations and CSF movement effecting glymphatic flow. Illustration of the proposed cascade of physiological events that combine to produce the slow oscillations known as *Intracranial B waves* or *CBF slow waves*, associated with CSF movement, that may provide a significant hydrodynamic driver of glymphatic flow. This proposed cascade was constructed as a possible explanation for the fact that B waves of the MCA velocity, and CBF slow waves, have both been reported in deep resting or sleep in normal individuals. They are also observed after, sedation, anesthesia, and other conditions that are associated with reduced level of consciousness including head injury. Both states are associated with delta wave EEG, with cyclic intermittent faster frequencies which appear to drive the CBF fluctuations (see also Table 2). In the awake state **(far left)**, multiple EEG frequencies and rhythms, including predominant alpha frequencies (8–12Hz), operate during the myriad of brain functions which occur simultaneously, when glymphatic flow is minimal. The level of consciousness decreases, **(center left)** during deep relaxation and non-REM (delta wave) sleep, after sedation or anesthetics, and following brain injury or global brain dysfunction, during various stages of coma. During decreased level of consciousness, the EEG shifts into a predominant delta wave (0–4Hz) baseline pattern, as inputs from deep brain regions including locus coeruleus reduce their excitatory input. During delta wave EEG predominance, intermittent oscillations of faster EEG activity including spikes and spindles occur at the same repeating cyclic frequency as reported for B waves measured by TCD (in cm/sec), and CBF slow waves measured by f-MRI (0.3–4 cycles per min, 0.005–0.067 Hz^*^) **(center right)** (with permission). These low frequency cyclic oscillations of faster electrical activity drive a repeating cycle of arterial vasodilatation and constriction at the same frequency. The resulting cerebral volume fluctuations, cause an accompanying localized hydrodynamic force, which may contribute to CSF movements **(far right)** from the periarterial CSF space through the brain interstitial space. These CSF pulsation movements can be measured in the ICP (mm/Hg) **(upper)**, and are indirectly observed using MRI imaging of CSF movement in the 4th ventricle **(lower)**. (With permission) ^*^Several reports indicate lower frequency values of 0.2 cycles per min using TCD, however the majority indicate lower frequency limits of 0.3 cycles per min.

The suggested driving forces for B waves have been vasomotor instability of the regulating vessels, or alternately a central nervous system driving mechanism for vascular fluctuations, which manifests as periodic cyclic fluctuations in EEG activity, at the B wave frequency (11, 16, 30). Variations in output signals from the brainstem noradrenergic neurons of the locus coeruleus to the cerebral cortex, may play a critical role in driving the periodic EEG activity fluctuations (31, 32) which are associated with B waves and CBF slow oscillations. In this scenario, during delta wave sleep, thalamocortical oscillations with lower EEG frequency (i.e. delta waves) become more dominant in the cortical EEG tracing (33, 34). Oscillations of episodic faster EEG activity that occur during delta wave sleep, including sleep spindles (35) and K complexes, have been referred to as “nested” frequencies (24, 36). These faster EEG transients, may combine to increase neuronal activation and overall brain metabolic demand, resulting in vasodilatation during the peaks of the intracranial B waves as well as during CBF slow waves (see Figure 6). Many of the detailed mechanisms of locus coeruleus modulation of this cascade however, are not completely understood.

Recently, autonomic arousals have been suggested as a possible candidate causing slow waves, acting through myoactive mechanisms to induce CSF pulsations (see also, second paragraph, limitations). In our recordings from intensive care patients, we found no relationship between heart rate variability (HRV) within the B wave frequency range and the existence of such large slow waves in ICP and FV. Moreover, the HR was practically the same in high as in low slow wave activity (Table 1). Assuming that HRV is an indicator of autonomous activity, the findings indicate that autonomic arousals is probably not involved in generating B waves in traumatic brain injury.

### Physiologic Role of Hemodynamic Oscillations, and Implications for CSF Flow and Glymphatic Drainag*e*

Fultz et al. (2) described a coherent pattern of oscillating electrophysiological activity measured by EEG spectroscopy, cerebrospinal fluid (CSF) movement in the 4th ventricle measured by MRI, along with oscillations in CBF measured by an fMRI BOLD sequence, in human volunteers. The transient higher EEG frequencies during delta wave background, preceded the BOLD sequence activation and the CSF peak flow by about 6–7 seconds, which is the established interval for neurovascular coupling (37, 38). The coupled physiological responses repeated at intervals of approximately 0.05 Hertz or 3 cycles per min (d/dt BOLD) in sleeping subjects but were absent or minimal in the awake state. The frequency range was not given. Reanalysis of our data in the subset of 29 head injury patients described above, indicated that the peak of the time derivative of B waves was 0.035 Hz in the d/dt of FV (see Figure 3). This is very close to the peak of the time derivative, of the d/dt 0.05 Hz of BOLD frequency, reported by Fultz et al. (2) (although; note that peak time derivative is skewed toward higher frequencies) (Table 2, Figure 5). The CSF movement measured in the 4th ventricle using MRI (2), was proposed as a driving force for glymphatic flow through the brain (39).

### Mechanisms and Significance of Glymphatic Flow

Circulation of the CSF has been studied extensively in the past (40, 41). Rennels et al. (42) described anatomical and physiological evidence in cats that CSF can circulate through the paravascular or Virchow Robin spaces around penetrating vessels and through the brain interstitial space. Subsequent studies have confirmed that arterial pulsations can facilitate perivascular CSF flow (43). Nedergaard, and colleagues have designated this pathway as the glymphatic system, demonstrating a role of glial cells, aquaporin 4 channels, the sleep-wake cycle, and clearance of amyloid-β during bulk flow in the interstitial space (44). Recent studies on glymphatic flow in rodents have demonstrated CSF tracer movement into deep brain regions, which may serve to deliver nutrients and clear waste products (45, 46), at markedly increased rates, in proportion to the level of sleep and EEG delta wave activity, suggesting a causal association.

Direct imaging of CSF contrast tracers also provides evidence in animals and in humans of glymphatic flow through the brain parenchyma. Hablitz et al. (47) described a rodent study of a fluorescent albumin-conjugated CSF tracer injected into the cisterna magna of anesthetized mice. The CSF tracer dispersion through the brain was strongly associated with concomitant delta wave EEG activity. MRI imaging in humans following administration of intrathecal CSF contrast material has also shown evidence of glymphatic flow, by contrast enhancement, beginning on the cortical surface, and then eventually reaching all brain regions (48, 49).

Kiviniemi et al. (5) have used ultra-fast MR encephalography to image CSF pulsations in the brain parenchyma in humans. These investigators were able to image brain pulsation responses to physiological drivers including cardiac pulsations, respiratory oscillations, and also other much slower spontaneous fluctuations that they termed as very low frequency(VLF; 0.01–0.027 Hz) or (0.6–1.6 cycles per minute), and low frequency oscillations (LF; 0.027–0.073 Hz) or (1.6–4.4 cycles per min) (5). These slower VLF and LF oscillations occur in the same frequency range reported for intracranial B waves (19) (Table 2, Figure 5).

Nedergaard and Goldman (1), presented evidence that the glymphatic system is activated during sleep, and can clear toxic solutes such as β-amyloid from the brain. A corollary of these observations is that poor sleep, and a consequently decreased duration of slow wave EEG activity and slow vasomotion, will result in reduced glymphatic flow and impaired clearance of metabolic products, potentially contributing to proteinopathies and dementia. The physiologic driving forces for CSF flow through the glymphatic pathway include arterial pulsations, respiration, and also slow vasomotion. The slow vasomotion described by Kivinemi et al. (5) comprised of VLF and LF waves at the frequencies shown in Table 2 and Figure 5, is becoming increasingly recognized as an important mechanism, driving glymphatic flow (1, 2, 39).

Taken together, these findings from animal experiments, as well as measurements of EEG, CBF, and CSF fluctuations in humans, indicate the presence of a remarkably consistent synchronized process. The cascade of electrophysiological fluctuations, intracranial B wave, or CBF slow wave activity, linked to CSF oscillations, occurs most actively during sleep or other causes of reduced level of consciousness. Figure 6 illustrates the proposed cascade of events, which has been linked together, from a comparison of the results of intracranial B wave measurements, MRI measurements, as well as other reported influencing factors.

### Terminology and Frequency Discrepancies

Rather than referring to B waves of the ICP, it may be more accurate to describe oscillations of CBF, cerebral blood volume, and CSF as “intracranial B waves”, or alternately as CBF and CSF slow oscillations. As previously reported, the amplitude of the CBF oscillations during B waves, appear to be just as robust in normal subjects as in head injured patients, however, due to the high intracranial compliance at normal ICP levels, these CBF oscillations are only associated with minimal changes in ICP. In a previous analysis we demonstrated that the amplitude of the ICP B waves was increased as a function of the mean ICP, i.e. lower intracranial compliance (3). Since Lundberg was not able to record CBF, or to apply spectral analysis of the B waves, he considered only ICP, which is the secondary effect of CBF oscillations. He therefore was only able to visualize a narrow frequency range 0.5–2 per min (10), rather than some of the lower and higher frequency CBF oscillations that are now detected by MRI and TCD methods.

### Limitations

The comparative analysis portion of this study was conducted using data which were collected previously, however, the recordings were stored and preserved in digital format with a sufficient sample frequency (50 Hz) and amplitude resolutions. As for ABP and CO_2_ measurements, the technology has barely changed. We also employed a state-of-the-art TCD machine with similar technology as used in current TCD monitoring applications. The epidural transducers used for ICP recordings did seem unreliable at very low (<5 mmHg) values, however these recordings were discarded as described in the methods section. We believe that for quantitative assessment of the B wave frequency spectrum and the cross-correlations with flow velocity, the quality of the ICP recordings were sufficient, even if the mean values might have been less accurate (50). The epidural ICP monitor(manufactured by Galetec, Ltd., Dunvegan, Scotland), was commonly used in Europe and the US before fiberoptic transducers became widely available, and had the safety advantage of not requiring dural penetration for insertion, and also stability of recordings over time.

It also should be noted that our comparison of physiologic variables in head injured patients, were all in an ICU setting in the supine position, with controlled ventilation. A recent report has indicated that in normal volunteers, autonomic events may occur which may also effect vascular tone and CSF movement with a slower lag time in comparison to those reported in this study (51). Analysis of these autonomic events may also be important as drivers of glymphatic flow, but detailed analysis of these factors was not in the scope of the present study. It is also possible that Lundberg C waves can contribute to CSF oscillations, however these generally occur at a higher frequency (0.1 Hz, or 6 x per min) and are thought to be generated by cardiac and blood pressure spontaneous and irregular frequency variations.

Regarding comparison of the RSx values between the fMRI data of the linear tracings of CBF slow wave intervals obtained from three available articles(*n* = 3), and the RSx values in our high B wave amplitude cohort(*n* = 31), it is acknowledged that the group size was small in the fMRI CBF slow wave cohort, due to the limited data displayed in this format, available for comparison. Despite this small sample size, the mean RSx values in the two groups were extremely close, providing support for the hypothesis that the wave generating mechanism for CBF slow waves and intracranial B waves share a similar degree of wave asymmetry.

Another consideration in comparing MCA velocity values to regional CBF values obtained from fMRI, is the possibility of regional variability in the CBF values. We recorded MCA velocity values, which supply the largest CBF distribution of all the regional cerebral arteries, however flow changes in the anterior and posterior cerebral arteries were not analyzed. Previous reports have also described velocity changes in other arteries at the same frequency as B waves however, supporting the idea that B waves may occur over a wider area than was reflected in these recordings (52).

## Conclusions

Reanalysis of TCD and ICP monitoring data, obtained in a previously reported cohort of head injury patients manifesting B waves, now reveals a similar frequency range, average frequency values, wave morphology, and latency to CSF fluctuations, when compared to similar parameters published on slow CBF and CSF oscillations derived from MRI studies. Both TCD with ICP monitoring, and MRI methods, describe similar physiologically coupled CBF and CSF oscillations, driven by EEG fluctuations, which are most prominently manifest during delta wave EEG predominance, occurring during sleep and reduced level of consciousness. The resulting cerebral blood volume oscillations produced by this cascade may serve as a pump to drive fluctuations of CSF and glymphatic flow, which is a key mechanism to clear the brain interstitial space of solutes, and for facilitating delivery of nutrients or medications to the brain parenchyma. These oscillations appear to drive an especially relevant mechanism for clearance of substances during sleep, and also may play a key repair role in conditions associated with impaired consciousness, including but not limited to, traumatic brain injury, stroke, intracranial hemorrhage, concussion, brain tumors, meningitis, neurodegenerative conditions, or other conditions characterized by abnormal fluid homeostasis in brain.

## Data Availability Statement

The raw data supporting the conclusions of this article will be made available by the authors, without undue reservation.

## Ethics Statement

The studies involving human participants were reviewed and approved by Department of Neurosurgery, University of Bern. The patients/participants provided their written informed consent to participate in this study.

## Author Contributions

DN and RA initial collection of patient data and study design for data presented on head injured patients. DN, MN, and RA for preparation of figures and critical contributions of writing and review of the manuscript. All authors contributed to the article and approved the submitted version.

## Funding

This work was initially funded by the William P. Van Wagenen Fellowship through the American Association of Neurological Surgeons.

## Conflict of Interest

The authors declare that the research was conducted in the absence of any commercial or financial relationships that could be construed as a potential conflict of interest.

## Publisher's Note

All claims expressed in this article are solely those of the authors and do not necessarily represent those of their affiliated organizations, or those of the publisher, the editors and the reviewers. Any product that may be evaluated in this article, or claim that may be made by its manufacturer, is not guaranteed or endorsed by the publisher.
